# The presence of *Helicobacter pylori* in oral cavities of patients with leukoplakia and oral lichen planus

**DOI:** 10.1590/1678-775720150203

**Published:** 2016

**Authors:** Magdalena Kazanowska-Dygdała, Irena Duś, Małgorzata Radwan-Oczko

**Affiliations:** Wroclaw Medical University, Department of Periodontology, Unit of Oral Pathology, Wrocław, Poland

**Keywords:** Helicobacter pylori, Leukoplakia, Lichen planus

## Abstract

**Objective:**

*Helicobacter pylori* infection is one of the most common bacterial infections in men. This gastrointestinal pathogen is closely related to gastritis, peptic ulcers, and the increased risk of gastric cancer. Numerous studies have indicated oral cavities as possible *Helicobacter pylori* reservoirs. *Helicobacter pylori* has been detected both in supragingival and subgingival plaques, and also in saliva. In addition, the relationship between lesions of oral mucosa and the presence of *H. pylori* has been evaluated and described in some studies. The aim of this study was to assess the presence of *Helicobacter pylori* DNA in the oral cavity of patients with oral leukoplakia and oral lichen planus.

**Material and Methods:**

The study included 54 patients with oral leukoplakia, 72 with oral lichen planus lesions, and 40 healthy controls. The presence of *Helicobacter pylori* in oral cavity samples was analyzed using a single-step Polymerase Chain Reaction (PCR) method. All patients underwent a periodontal examination and the following clinical parameters were collected: pocket depth, bleeding, and plaque indexes. The periodontal status was assessed using the Offenbacher classification.

**Results:**

In most patients, pathological lesions were in typical sites on the buccal mucosa (leukoplakia in 88%, and oral lichen planus in 93% of patients). The DNA of the *Helicobacter pylori* was present in 20% of patients with leukoplakia and 23% of patients with lichen planus. We did not find the DNA of *H. pylori* in healthy controls. The periodontal status described by periodontal indices was worse in the investigated group than in the control group.

**Conclusion:**

These findings suggest that the *H. pylori* presence in oral cavities may be related with leukoplakia and lichen planus oral lesions.

## INTRODUCTION

The *Helicobacter pylori* (*H. pylori*) is one of the most common and well-known bacterium in the world. It colonizes the human stomach and it is responsible for chronic gastritis, peptic ulcer disease and, recently, has been recognized as a risk factor for gastric adenocarcinoma. The *H. pylori* infection is considered a serious transmissible disease. The exact way of transmission of these bacteria is still debated, although some evidence suggests that it is most likely to happen during a direct person-to-person contact^6^. Apart from its presence in the stomach, the *H. pylori* has also been found in dental plaque and feces. Hence, it is thought that the route of infection can be oral-oral or fecal-oral^6^. The role of dental plaque as a reservoir of *H. pylori* and a possible source of infection or reinfection of gastric mucosa has been discussed for a long time. Some studies indicate a relation between the infection of *H. pylori* in oral cavities and stomachs, but observations concerning the role of oral cavity as another niche for *H. pylori* are controversial^27^. In about 40% of patients with gastritis, the bacterium is also present in the oral cavity, which may indicate the transient character of the infection. Nevertheless, it is also suggested that *H. pylori* may be present in the oral environment as normal bacterial flora^22^. *H. pylori* has been detected both in supragingival and subgingival plaques, and in saliva with and without a concomitant stomach infection^11^ ^,^ ^14^.

Oral Lichen Planus (OLP) is a common T-cell-mediated chronic inflammatory oral mucosa disease of an unclear etiology, despite the hypothesis that antigen-specific and non-specific mechanisms are involved^19^ ^,^ ^23^. Possible etiological factors are: viral infections, mental stress, mechanical trauma, and individual susceptibility probably related to genetic predispositions^20^ ^,^ ^26^. It has typical bilateral localization mainly on the posterior buccal mucosa and on the lateral margins of the tongue, and its clinical presentations range from a white reticular plaque to an atrophic, erosive and rarely of bullous form. In the reticular form, this lesion can often be asymptomatic. However, red forms are painful and spicy or acidic food and mechanical irritations exacerbate unpleasant sensations^7^ ^,^ ^24^.

Oral leukoplakia is a well-known potentially malignant lesion. Based on its etiopathogenesis, there are two different forms of this white lesion – one idiopathic and the other one associated with tobacco use. Clinically, two types are present: homogeneous and non-homogeneous leukoplakia. Although leukoplakia can vary histopathologically, its features include hyperkeratosis, orthokeratozis or parakeratozis, acanthosis of the epithelium, and chronic inflammatory infiltrations into the lamina propria. Also, various degrees of epithelial dysplasia may be seen but only in the minority of lesions^9^ ^,^ ^25^.

Leukoplakia and oral lichen planus are frequent oral mucous lesions. Although both pathologies have been deeply researched, there is a lack of unequivocal observations concerning the relation of these diseases with a *Helicobacter pylori* infection*.*

The aim of the present study was to assess the presence of the *H. pylori* DNA in the oral cavity of patients with mucosal pathologies, such as leukoplakia and oral lichen planus, and to examine the hypothesis of coexistence of bacteria in the investigated oral lesions.

## MATERIAL AND METHODS

All patients provided informed consents before taking part in the study. There were three groups: with oral leukoplakia (group L), with oral lichen planus (group OLP), and the control group. About 54 subjects (30 females and 24 males) comprised the group L and 72 (55 females and 17 males) the OLP group without any gastrointestinal problems. The control group consisted of 40 generally healthy patients without lesions in the oral cavity. All patients with leukoplakia were current smokers with no cell dysplasia in the histological examination. In the group with OLP, only the reticular or reticular-erosive forms were present. The diagnosis of oral lichen planus was made according to the modified Word Health Organizations (WHO) diagnostic clinical criteria^24^.

Exclusion criteria for the study were: general antibiotic treatment within three months prior to the examination, patients with severe P3 periodontitis (PD≥4 mm, BOP extent score ≥50%), according to the Offenbacher classification^15^.

The age, the number of teeth, smoking habit, and the site of the oral lesions were collected from all patients. A complete oral/periodontal examination was performed by a single dentist; Pocket Depth (PD) was measured with a periprobe at 4 sites per tooth; oral hygiene was evaluated according to the Approximal Plaque Index (API)^12^, and the bleeding was according to the Bleeding on Probing (BOP)^2^; the periodontal status was assessed according to a simplified periodontal disease classification of Offenbacher, et al.^15^ (2008). To evaluate the presence of *H. pylori* DNA, two samples were taken from the premolar/molar interdental spaces or from the periodontal pockets, depending on the stage of periodontium. The area around the chosen sites was dried and isolated from saliva. Then, a sterile microbrush was inserted into the space/pocket, transferred to a sterile Eppendorf tube, and frozen at -20°C.

### DNA extraction protocol

Laboratory tests were carried out entirely at the Department of Molecular Techniques of the Wrocław Medical University. Each of the micro brushes with the collected material was placed in a 1.5 ml Eppendorf tube. Four hundred milliliters of sterile water (Sigma Aldrich Reagent Water Molecular Biology) was then added to the tubes and vortexed. DNA was isolated using the modified Hexadecyl Trymetyloammonium Bromide (CTAB) method.

### PCR method

To confirm the accuracy of the sample collection and the effectiveness of the DNA isolation method, the internal control reaction for the presence of human beta-actin genes was carried out. The reaction was conducted with the use of a single-step PCR (GeneAmp^®^ PCR System 9700, Applied Biosystems, Waltham, Massachusetts, USA).

The *H. pylori* DNA amplification technique was performed properly with the use of located (nested) PCR. It consists in the carrying out of two, one after another, PCR reactions using two different pairs of suitable and specific primers to the genomic DNA of bacteria. The described type of nested PCR reaction is based on two stages. Next, we show the composition of the reaction mixture for two successive PCR for a DNA sample. In the first reaction mixture, to 6.05 ml of H_2_O water (Gibco, Invitrogen, Paisley, Scotland, UK) we added 1.0 ml of PCR developing buffer, 0.5 mL of dNTP at a concentration of 10 μM, 0.25 ml of a mixture of primers EHC at a concentration of 10 μM, and 0.2 ml of Taq polymerase (reaction buffer for DSF-Taq-Taq pol.Top, Bioron, Ludwigshafen am Rhein, Germany). In this reaction, 2 μl of DNA extracted from oral cave was added. A set of primers used in the first reaction (EHC-U/-L) was chosen for the 860 bp fragment of *H. pylori* genomic DNA (80 076-80 492 bp). In the second reaction, 7.65 ml of DNase-free water was added, 1.0 ml of PCR buffer, 0.5 μl of DTP with a concentration of 10 μM, 0.25 μl starters that was named ET after nomination given by Song, et al.^22^ (2000) with a concentration of 10 μM and of 0.1 Taq polymerase (5000 U/ml). In the second reaction, we added 0.5 μl of the DNA amplified in the first reaction. The second set of primers (ET-5U/ET-5L) was internally directed to the sequence of 860 bp fragment of *H. pylori* genomic DNA amplified by the previously used primers (EHC-U/EHC-L)^22^.

**Figure 1:**
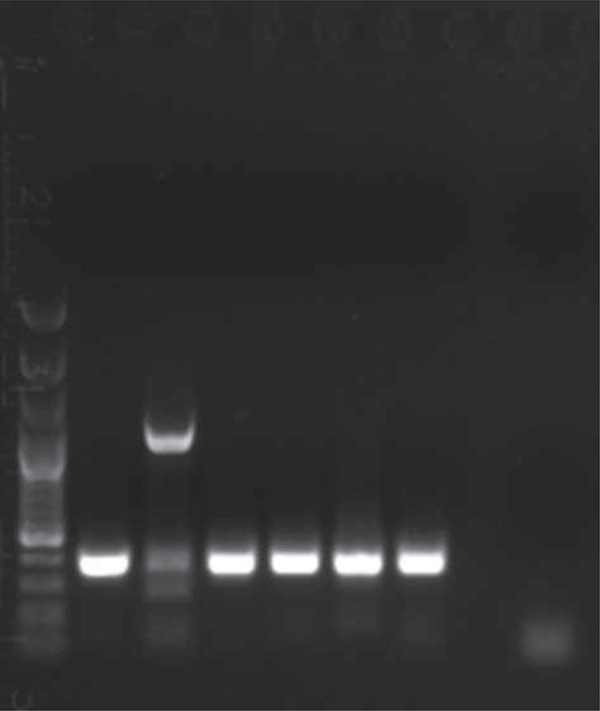
Results of positive control reactions for the correct DNA isolation from oral caves - beta actin gene. Description of results: 1- Gene Ruler 100 bp Ladder (Fermentas); 2- Probe n. 9; 3- Probe n. 10; 4- Probe n. 11; 5- Probe n. 12; 6- Probe n. 13; 7- Probe n. 14; 8- Negative control (H2O instead of DNA)

**Figure 2:**
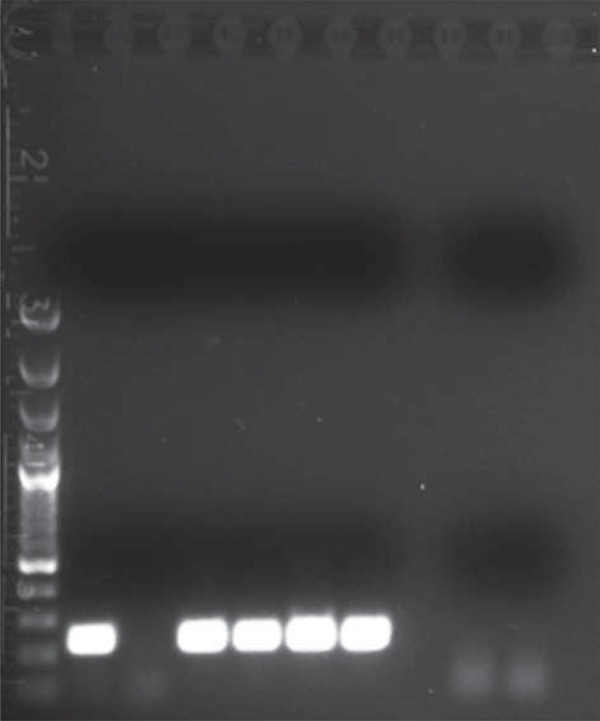
Detection of *Helicobacter pylori* with nested Polymerase Chain Reaction (PCR) of the second PCR reaction. Description of results: 1- Gene Ruler 100bp Ladder (Fermentas); 2- Probe n. 9; 3- Probe n. 10; 4- Probe n. 11; 5- Probe n. 12; 6- Probe n. 13; 7- Probe n. 14; 8/9: Negative control (H2O instead of DNA)

To visualize the PCR products, electrophoresis was carried out in a 1% agarose gel in the TAE mixture (Tris 242 g, 100 ml of 0.5 M EDTA pH 8; 57.2 ml CH_3_COOH *per* 1000 ml) in the presence of ethidium bromide (a concentration of 10 mg/ml). For the number of base pairs, we used a DNA GeneRuler 100 bp DNA Ladder (Fermentas, Thermo Fisher Scientific, Waltham, USA) as a DNA size standard. Visualization of electrophoresis results was performed under UV light with the use of the Kodak Gel Logic 100 Imaging System (Eastman Kodak Company). Examples of both *H. pylori* and beta actin reactions are presented in Figures 1 and 2.

### Statistical analysis

The results of the research were statistically analyzed. The following parameters were calculated for all groups: the number of cases (N), average values (X), the median (M), the range (min-max), the upper and lower quartile (25Q-75Q), and standard deviations (SD).

Verification of the hypothesis of the equality of each average trial was conducted using the ANOVA method (variance analysis). The Kruskal-Wallis rank sum test for nonparametric data was carried out in the groups in which there was heterogeneous variance. We recognized P≤0.05 as statistically significant. The analysis was conducted using the EPIINFO 7.1.1.14 statistical software (dated 2-07-2013).

## RESULTS

We summarized the clinical characteristics of the investigated and control groups in Table 1.

**Table 1:**
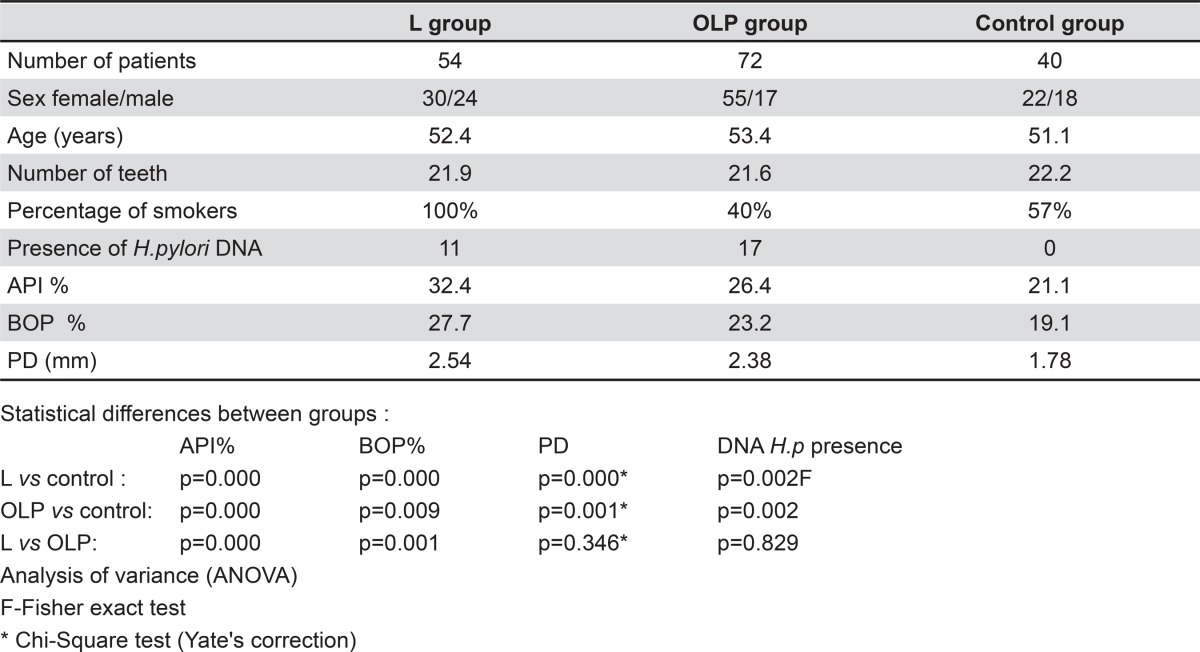
Characteristics of patients

We found no differences about the average age and the number of teeth between the groups of patients with leukoplakia and lichen planus, and the control group. Fifty-four patients from the leukoplakia group, 29 patients from the OLP group, and 23 from the control group were smokers.

Pathological lesions were mainly present in the typical sites of the buccal mucosa (in 89% of patients with leukoplakia and in 93% of patients with oral lichen planus). There were no differences in the presence of *H. pylori* DNA between the investigated groups. We found this pathogen in 20% of patients with leukoplakia and in 23.6% of patients with lichen planus. We found a significantly higher incidence of *H. pylori* DNA in both groups with oral pathologies in comparison to the control group (p=0.002), in which we did not detect the DNA of such pathogen (Table 1).

The average value of the API index was statistically higher both in the leukoplakia group in comparison to the lichen planus group (p=0.000) and the control group (p=0.000), and in the OLP group versus the control group (p=0.000).

Similarly, the average value of the BOP index was statistically higher both in the leukoplakia group in comparison to the control group (p=0.000) and the OLP group (p=0.001), and in the OLP group and the in comparison to control group (p=0.009).

The average pocket depth was statistically deeper in the leukoplakia group as well as in the OLP group in comparison to the control group.

By only comparing the two study groups according to the periodontal status, we found the oral hygiene was statistically worse and gingival bleeding was greater in patients with leukoplakia. However, there was no difference in the depth of the periodontal pocket (p=0.346).

On the other hand, the periodontal status described by the API, BOP, and PD indexes, in both of the investigated groups, was statistically worse in comparison with the control group, in which we did not detect the DNA of *H. pylori* (Table 1).

Furthermore, in the group with leukoplakia, there were statistically higher values for the API, BOP, and periodontal PD indexes in these patients, in which we found the presence of *H. pylori* (Table 2). Conversely, in the OLP group, whose percentage of bacterial DNA was the highest, the only statistically significant difference between patients with and without the presence of *H. pylori* DNA was in the periodontal PD (2.88 mm and 2.22 mm respectively, p=0.003). Although the average PD was greater in patients harboring the DNA of such bacteria, it was not described as related with periodontitis (Table 2).

**Table 2:** Comparison of periodontal indices average values in OLP and L groups in patients with (+) and without (-) the presence of *H. pylori* DNA

	*H. pylori* +	*H. pylori* -	Statistical importance
GROUP OLP	N=17	N=55	
API	26.4%	26.5%	p=0.947
BOP	25.5%	22.5%	p=0.139
PD	2.88 mm	2.22 mm	p=0.003
GROUP L	N=11	N=43	
API	35.5%	31.7%	p=0.030
BOP	32.4%	26.5%	p=0.024
PD	3.36 mm	2.33 mm	p=0.000

Non-parametrical Kruskal-Wallis test

API=Approximal Plaque Index

BOP=Bleeding on Probing

PD=Pocket Depth

## DISCUSSION

The relationship between lesions of the oral mucosa and the presence of *H. pylori* has been evaluated and described in a limited number of studies. An association between gastrointestinal diseases and aphthae (oral ulceration) has been demonstrated and the interest around this pathology and a *H. pylori* infection aroused. Albanidou-Farmaki, et al.^3^ (2005) stated that 34 out of 48 patients with recurrent minor aphthous ulcerations tested positive for *H. pylori.* He detected the assessed protein antibodies IgG, IgA and anti-CagA in the serum of 59 patients and in the saliva of 24 patients. The authors also found that the appropriate eradication of the bacteria led to the complete cure of the lesions or to remarkable improvements. In another investigation, we observed the presence of IgG antibodies against *H. pylori* in 52% of the investigated patients with recurrent aphthous stomatitis (RAS). However, the authors found *H. pylori* DNA in the ulcerations of only one case and concluded that this pathogen is not related with RAS^13^. Riggio, et al.^18^ (2000), using the PCR, found *H. pylori* DNA in 3 out of 28 biopsies from RAS^18^. Based on the results of 22 patients with various mucosal ulcerative disorders (including recurrent aphthous stomatitis, virus herpes simplex lesions, and erosive lichen planus lesions), Shimoyama, et al.^21^ (2000) suggested that there is no association between these pathologies and the presence of *H. pylori*. Only two out of seven patients with a herpes simplex infection tested positive for *H. pylori* – both for the culture test and for the *H. pylori* antigen^21^. In an earlier investigation, Porter, et al.^16^ (1997) examined serum IgG antibodies and did not find a *H. pylori* infection as an essential etiologic factor in the development of recurrent aphthous stomatitis and other oral mucosal lesions with ulcerations^16^.

The etiology of oral leukoplakia and oral lichen planus has not been fully explained. In this study, lesions were mainly in the buccal mucosa [leukoplakia in 48 (89%) patients and in 67 (93%) patients with OLP]. We found a significantly higher incidence of *H. pylori* DNA in the samples of the oral cavity of patients from both study groups -22.2% in comparison to the control group, in which the bacteria was not present. However, there were no statistical differences between the presence of *H. pylori* DNA in the group with leukoplakia -20% and with lichen planus -23.6%.

There are no studies on the presence of *H. pylori* DNA in oral cavity of patients with leukoplakia. In a recent study carried out in the Southwestern of Iran with 41 patients, researchers detected the IgG anti *H. pylori* in as many as 52% of oral lichen planus patients and 66% of control group patients. The authors did not find an association between a *H. pylori* infection and OLP, regardless of the clinical presentation^17^.

There is ambiguous view as to whether or not the oral cavity may harbor *H. pylori,* particularly in periodontitis patients. By evaluating 30 adult patients positive to a urease test with diagnosed gingivitis or periodontitis, Gebara and coauthors^10^ (2004) found *H. pylori* in saliva, in the supragingival and subgingival plaque of 43.3% of subjects, and a lack of statistically significant association with the patients’ periodontal status^10^. Other authors also failed to find a significant association between periodontal disease and poor oral hygiene and a *H. pylori* infection^4^ ^,^ ^5^.

However, based on the study of a cross-sectional data analysis by Dye, et al.^8^ (2002), it was found that poor periodontal health related with advanced periodontitis chracterized by deep periodontal pockets may be associated with a *H. pylori* oral infection.

In this study, the oral hygiene and the periodontal status of the study groups with leukoplakia and OLP were statistically worse in comparison to the control group. This may indicate the association between the presence of *H. pylori* DNA and dental plaque, and also the association between the inflammation of periodontal tissues and the presence of *H. pylori* could be taken into consideration. The worse periodontal status and oral hygiene were also present in *H. pylori* positive patients with leukoplakia. In *H. pylori* positive patients with OLP the only significant difference was in the depth of the periodontal pocket. Although the levels of oral hygiene and gingival bleeding were worse in the group with leukoplakia, we saw no statistical difference in the presence of *H. pylori* DNA between these groups. We should also highlight the average periodontal pocket depth (3.36 mm in leukoplakia and 2.88 mm in OLP *H. pylori* positive patients) was not related to periodontitis. Some studies underline the relationship between the presence of *H. pylori* and the occurrence of a periodontal pocket with a depth of 5 mm or more and advanced periodontitis^1^. Results of this study indicated the presence of *H. pylori* was not related with periodontitis.

In our study, we selected patients according to the periodontal status, which was characterized by mild periodontitis according to the Offenbacher classification. We excluded patients with periodontal pockets deeper than 4 mm and with BOP greater than 50% from the study. The current study shows the association between the presence of *H. pylori* DNA and pathological oral mucosa lesions such as leukoplakia and oral lichen planus. Since the evaluated state of periodontium and hygiene levels were worse in patients with *H. pylori* DNA, the influence of *H. pylori* on the development of these pathologies is debatable. On the other hand, the presence of *H. pylori* may have an additional influence on the local environment. Considering the role of *H. pylori* in pathogenesis of oral mucosal lesions or ulcerations is still unclear, it seems that patients with oral lesions as leukoplakia and oral lichen planus, and concurrent gastric problems, should be tested for the presence of a *H. pylori* infection.

It is important to take care of patients who need therapeutic interventions to improve their oral hygiene and periodontal status and eliminate any possible additional factors in the etiology of these lesions.

## CONCLUSION

The results of this study show the higher *H. pylori* presence in the oral cavity of subjects with leukoplakia and oral lichen planus when compared with control group. The described findings indicate that further research on this topic is necessary.
